# Kissing stent management of stenosis of two branches of left renal artery bifurcation: a case report

**DOI:** 10.1186/s13256-019-2119-3

**Published:** 2019-06-28

**Authors:** Abdala M. Mkangala, Henry A. Mayala, Khamis H. Bakari, Xiang J. Dong

**Affiliations:** 1 Department of Radiology, Wuhan Union Hospital, Tongji Medical College, Huazhong University of Science and Technology, 1277 Jiefang Road, Wuhan, 430022 China; 2 Department of Cardiology, Wuhan Union Hospital, Tongji Medical College of Huazhong University of Science and Technology, 1277 Jiefang Road, Wuhan, 430022 China

**Keywords:** Secondary hypertension, Kissing stent, Renal artery stenosis

## Abstract

**Background:**

Secondary hypertension accounts for 5% of all cases of hypertension. Renal artery stenosis is one of the common causes of secondary hypertension. Atherosclerosis and fibromuscular dysplasia are the commonest types of stenosis associated with renal vascular hypertension, with the former accounting for 70–80% of all cases and the latter accounting for 10% of the incidence. The greatest incidence atherosclerosis is in men over the age of 40 years, mostly affecting the proximal part of the renal arteries, whereas fibromuscular dysplasia affects women ranging in age from 30 to 50 years. Currently, possible treatments are medical treatment using blood pressure-lowering drugs, balloon angioplasty with or without stent insertion, and surgery to reconstruct the artery.

**Case presentation:**

We report a case of a 46-year-old Asian woman with stenosis of two branches of renal artery bifurcation treated by percutaneous balloon dilatation and stenting of both branches after referral to our department for a renal angiogram following 8 months of uncontrolled hypertension despite receiving medications. Initially, the patient presented with severe headache and fatigue. She was a known nonsmoker, was not diabetic, and had no history of diabetes in her family. She had no history of atherosclerosis. Apart from high blood pressure, the result of her physical examination was unremarkable. Laboratory investigations revealed normal serum cholesterol, lipid profile, and serum creatinine. She had been attending a hypertension clinic and receiving antihypertensive drugs for the past 8 months on a regular basis under close observation. Despite this treatment and care, her blood pressure remained high at 175/110 mmHg, which the attending doctor concluded to be uncontrolled blood pressure. Initial imaging indicated left renal artery stenosis, and the patient was referred to our department.

**Conclusions:**

For patients with uncontrolled hypertension despite receiving medications, renal Doppler ultrasound should be included in the diagnostic workup for secondary hypertension. Once renal artery stenosis is suspected, renal angiography is highly recommended because the technique is able to accurately diagnose stenosis in the branch arteries, unlike computed tomographic angiography and magnetic resonance angiography. Percutaneous transluminal renal angioplasty is the treatment of choice for renal artery stenosis in patients with renovascular hypertension or renal dysfunction.

## Background

Secondary hypertension account for 5% of all cases of hypertension. Renal artery stenosis is one of the common causes of secondary hypertension [1–3]. Atherosclerosis and fibromuscular dysplasia are the commonest types of stenosis associated with renal vascular hypertension (RVH). Atherosclerotic renal artery stenosis (ARAS) is the commonest type and accounts for 70–80% of all cases. The greatest incidence is in men over the age of 40 years, mostly affecting the proximal part of the renal arteries [4] .Unlike ARAS, renal artery fibromuscular dysplasia (RAFMD) affects women ranging in age from 30 to 50 years; among patients with RVH, the incidence of RAFMD is about 10%.

Angioplasty with or without stenting is the preferred choice of treatment for both lesions. Apart from the effect of RVH, azotemic renovascular disease, also known as ischemic nephropathy, is an indication for angioplasty [5]. Percutaneous transluminal balloon angioplasty is an accepted treatment for selected cases of renal artery stenosis caused by fibromuscular dysplasia [3], and stenting is the primary endovascular means for the treatment of ARAS, with technical success rates for renal artery stent placement approaching 95% [6].

We report a case of a patient with stenosis of stenosis of two branches of left two branches of renal artery bifurcation treated by percutaneous balloon dilatation and stenting of both renal artery branches. The angioplasty technique for this procedure in the management of stenosis of both branches of renal artery bifurcation is also discussed.

We decided to write this case report because our patient was different from previously reported patients in that there were no cardinal features of atherosclerotic or fibromuscular renal artery stenosis apart from bifurcated stenosis, but the patient did not respond to usual balloon angioplasty despite repeated application of balloon angioplasty, hence making our case report of interest so that other clinicians can learn from it.

## Case presentation

### Clinical history

**Fig. 1:**
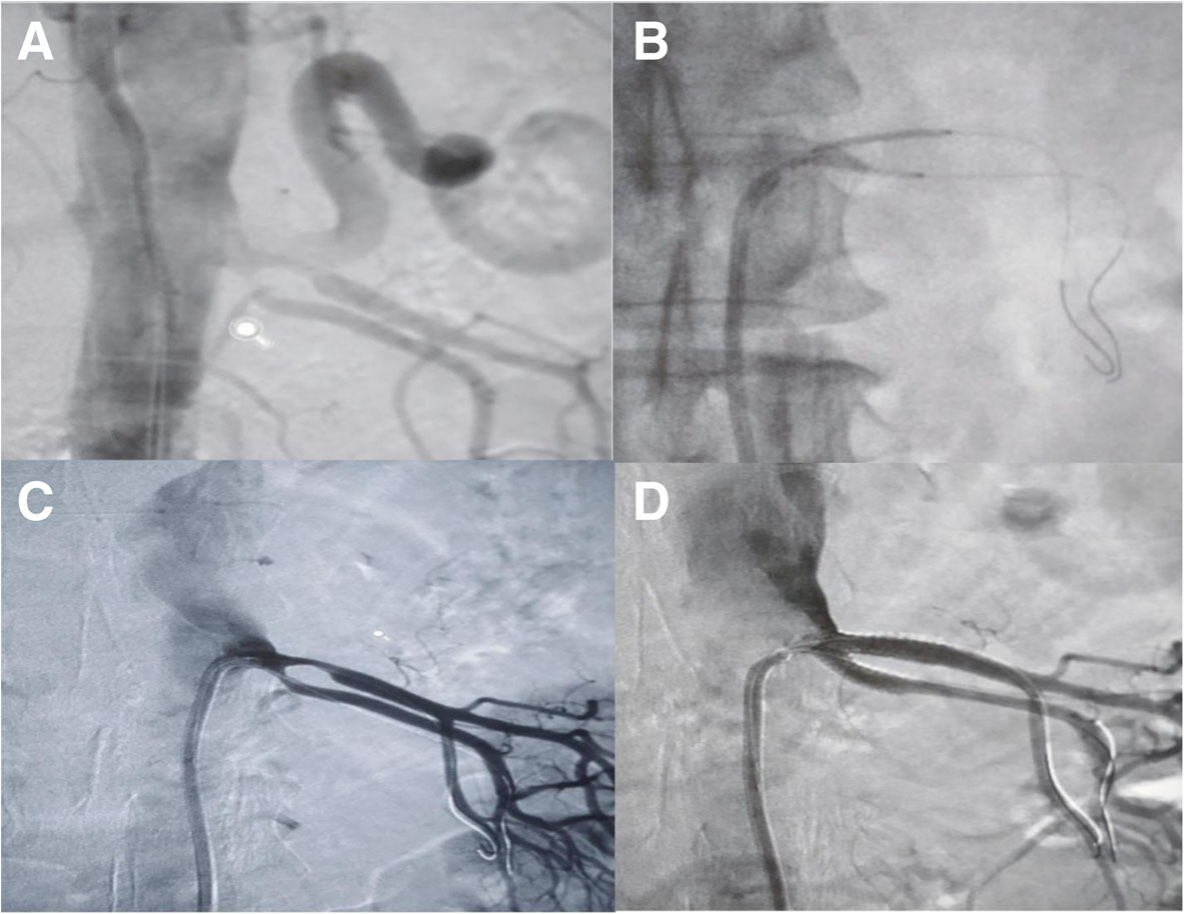
**a** Aortogram. **b** Placement of balloon in upper and lower branches, respectively, through the same vascular sheath. **c** Post-balloon arteriogram shows persistence of stenosis. **d** Post-stenting arteriogram shows improvement of lumen and good flow on both branches

A 46-year-old Asian woman was referred to our department for a renal angiogram following 8 months of uncontrolled hypertension despite receiving medications. Initially, the patient presented with severe headache and fatigue. She had no history of smoking or drinking alcohol, was not diabetic, and had no history of diabetes in her family. She had no history of atherosclerosis. Apart from high blood pressure, the result of her physical examination was unremarkable; her general, cardiovascular system, respiratory system, and abdominal examinations were unremarkable. Neurological examination on admission showed that the patient was alert, attentive, and oriented. Her speech was clear and fluent with good repetition, comprehension, and naming. She recalled 3/3 objects at 5 min. All of her cranial nerves were intact. Motor examination revealed no pronator drift of outstretched arms. Her muscle bulk and tone were normal. Her strength was full bilaterally. Her reflexes and sensory were both intact. Her coordination and gait were normal. Laboratory investigations revealed normal complete blood count, serum cholesterol, lipid profile, and renal function (serum creatinine 119 μmol/L). Her left kidney size was normal with measurement of 9.6 cm by 4.8 cm. Renal Doppler ultrasound confirmed renal artery stenosis with renal resistive index of 0.58. The percentage of renal artery stenosis in the two branches of the left renal artery was 70% and 75%, respectively , before the first balloon angioplasty; after the first balloon angioplasty, these percentages remained the same. After the second ballooning and stenting procedure, revascularization was achieved. The patient had been attending a hypertension clinic and receiving antihypertensive drugs for the past 8 months on a regular basis under close observation. Despite this treatment and care, her blood pressure remained high at 175/110 mmHg, which the attending doctor concluded to be uncontrolled blood pressure. Initial imaging indicated left renal artery stenosis, and the patient was referred to our department (Fig. 1). Prior to the diagnosis of renal artery stenosis, the patient had been receiving amlodipine 10 mg twice daily, bisoprolol 10 mg twice daily, and indapamide 2.5 mg every morning.

### Endovascular procedure

The procedure was performed under the guidance of digital subtraction angiography (floor-mounted Artis zee; Siemens Medical Solutions, Munich, Germany) using the Seldinger technique. With the patient under local anesthesia, the right femoral artery was punctured by a 21-gauge vascular access needle with an angled tip 0.035-inch guidewire, then catheterized with a 5-French introducer sheath (Terumo Interventional Systems, Tokyo, Japan). The first aortogram was obtained using a pigtail catheter (Fig. 1a), then an 8-French guiding catheter (Cook Medical, Bloomington, IN, USA; Cordis, Hialeah, FL, USA) was used to obtain selective renal angiograms whereby the proximal main flow and the stenosis of both branches and their respective distal flow on the left renal side were revealed. The right renal artery was normal in appearance. The left renal artery angiogram then was used as a reference for further guided interventional procedures in which the individual length and diameter of stenosis were measured. The decision was reached to perform percutaneous transluminal renal angioplasty, and the length and diameter of balloon needed were calibrated. With two balloons of 4 mm × 18 mm (Biotronik, Berlin, Germany), both were was dilated at the same time. Despite expert effort in dilatation, the stenosis was observed to persist (Fig. 1c). Stent placement was considered, and the procedure was continued. A preprocedure intravenous bolus of 5000 IU of heparin was administered. By using two 0.014-inch guidewires (V14; Boston Scientific, Natick, MA, USA), the interventional radiologist guided the stent to cross the upper and lower branches, respectively, through the same vascular sheath **(**Fig. 1b). Two balloon expandable stents measuring 4 mm × 18 mm and 5 mm × 18 mm (Biotronik) were placed in parallel (kissing) and simultaneously inflated both branches. A good angiographic result was revealed (Fig. 1d) with no need for further ballooning. Angiography contrast media (Omnipaque 350; GE Healthcare, Shanghai, China) were used. Volumes of 25 ml of contrast agent were injected at a flow rate of 5 ml/s. The final angiogram was obtained to confirm the position of the stent, the patency of the lumen, and distal blood flow. Finally, the femoral access site was closed with Perclose ProGlide (Abbott Vascular, Chicago, IL, USA). After the procedure, the patient was admitted in the ambulatory room for further observation. Her blood pressure was monitored and recorded, it showed a significant reduction of blood pressure to 128/87 mmHg. After 24 h of observation, the patient was discharged to home with aspirin (100 mg/day) and clopidogrel (75 mg/day for 3 months). During 12 months of follow-up, the patient remained well with blood pressure of 126/87 mmHg. Renal ultrasound showed bilateral kidneys of normal size and shape with good cortical medullary differentiation. A bilateral renal Doppler study appeared normal.

## Discussion

**Fig. 2:**
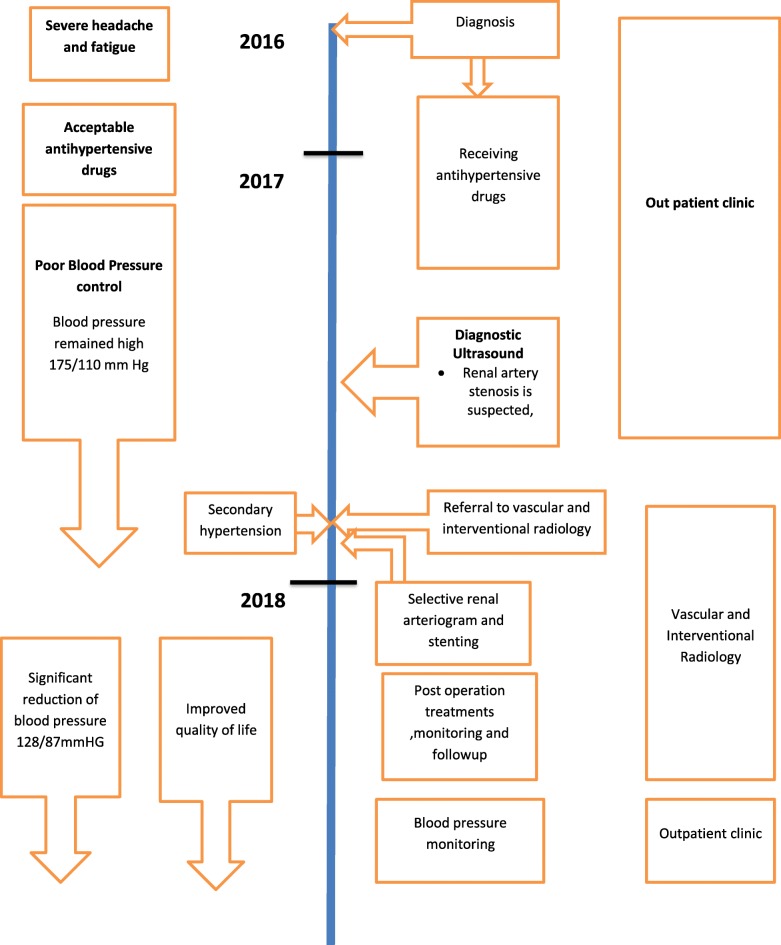
Timeline of interventions and outcomes

We report a case of a 46-year-old woman with stenosis of two branches of renal artery bifurcation treated by percutaneous balloon dilatation and stenting of both branches after referral to our department for a renal angiogram following 8 months of uncontrolled hypertension despite receiving medications (amlodipine 10 mg twice daily, bisoprolol 10 mg twice daily, indapamide 2.5 mg every morning) (Fig. 2). We decided to write this case report because our patient was different from previously reported patients in that there were no cardinal features of atherosclerotic or fibromuscular renal artery stenosis, making our case report appealing and interesting so that other clinicians can learn from it.

Renal artery stenosis is one of the common causes of secondary hypertension [1–3]. Atherosclerosis and fibromuscular dysplasia are the commonest types of stenosis associated with RVH. Severe stenosis may lead to loss of excretory function of the kidney. Despite its prevalence, ARAS is poorly defined. Its incidence is approximated to range from 30% among patients with coronary artery disease detected by angiographic study to 50% among elderly people or those with diffuse atherosclerotic vascular diseases [7]. Atherosclerotic lesions are primarily ostial or proximal in location. ARAS is a progressive disease that may occur alone or in combination with hypertension and ischemic heart disease, unlike in our patient [8]. Atherosclerotic lesions respond poorly to balloon angioplasty alone with significant residual stenosis and restenosis. Stent placement in this case is highly recommended because it has been shown to improve immediate and long-term outcomes [8–10].

Currently, possible treatments are medical treatment using blood pressure-lowering drugs, balloon angioplasty with or without stent insertion, and surgery to reconstruct the artery. Percutaneous transluminal balloon angioplasty is widely acceptable management for selected cases of renal artery stenosis caused by fibromuscular dysplasia [3]. Stent placement can play a very integral role in therapy for patients with lesions difficult to treat with balloon angioplasty, as well as after a suboptimal balloon angioplasty result. Surgical reconstruction of the renal artery is generally performed only in patients with complicated renal artery anatomy or in those who require pararenal aortic reconstructions for aortic aneurysms or severe aortoiliac occlusive disease. Despite expert effort to dilate the stenosis, it was observed to be persisting, unlike in ARAS reported in the CORAL (Cardiovascular Outcomes in Renal Atherosclerotic Lesions) trial, in which there were not any clinical benefits for hypertension and chronic kidney diseases, which was also reported in another study by the Angioplasty and Stenting for Renal Artery Lesions (ASTRAL) investigators [11, 12]. Our patient’s case was different in that there were no cardinal features of atherosclerotic or fibromuscular renal artery stenosis, making our case report appealing and interesting so that clinicians can learn from it. Although endovascular procedure is successful in the treatment of ARAS, one should be aware that this is a local complication of a systemic disease. Thus, patients should be given secondary preventive measures, including statins, platelet inhibitors, and antihypertensive therapy [13].

## Conclusions

For patients with uncontrolled hypertension despite receiving medications, renal Doppler ultrasound should be included in the diagnostic workup for secondary hypertension. Once renal artery stenosis is suspected, though computed tomographic angiography or magnetic resonance angiography is often warranted, neither of these two techniques is able to accurately diagnose stenosis in the branch arteries; hence, renal angiography is highly recommended. Renal angiography is suitable for diagnosing renal stenosis and allows further intervention in which percutaneous transluminal renal angioplasty can be performed in the same setting. Percutaneous transluminal renal angioplasty is the treatment of choice for renal artery stenosis in patients with RVH or renal dysfunction. However, this is invasive treatment, and one should consider the indications and contraindications.

## Abbreviations

ARAS: Atherosclerotic renal artery stenosis
RAFMD: Renal artery fibromuscular dysplasia
RVH: Renal vascular hypertension
